# Novel digital measurement system for predicting surgical outcomes in patients with primary non-syndromic craniosynostosis

**DOI:** 10.1016/j.jobcr.2025.01.025

**Published:** 2025-02-21

**Authors:** Andrea Grandoch, I Mohammed Barham

**Affiliations:** a Department for Oral and Craniomaxillofacial and Plastic Surgery, Faculty of Medicine and University Hospital Cologne, University of Cologne, Germany; b Department II of Anatomy, Faculty of Medicine, University Hospital Cologne, Cologne, Germany

## Abstract

**Objective:**

With the aim of further optimizing the care of patients with primary non-syndromic craniosynostosis, we describe a novel and clinically feasible measurement method to predict postoperative outcomes and provide an analysis of quality of life.

**Design:**

76 patients with primary non-syndromic craniosynostosis were treated by one surgeon. 47 healthy patients without craniosynostosis formed the control group. All patients had an age between 3 months and 18 years.

Based on manual measurement using callipers, x-ray-imaging and 3-D-photographs of the head, various detailed symmetry and aesthetic indices were collected using a novel digital measurement tool that was integrated into a clinically established programme. These are compared with a healthy control group without craniosynostosis. In addition, perioperative data, a clinical visual assessment of the scars and quality of life were evaluated using a standardised questionnaire.

**Results:**

Individual values show statistically significant deviations from the control group preoperatively and immediately postoperatively, which are typical for the respective form of craniosynostosis. Overall, there were good results in terms of symmetry, aesthetics and satisfaction. Interestingly, the quality of life of operated patients tended to be rated better overall than in the control group.

**Conclusion:**

The detailed measurement technique presented is easy to use and enables an individual, efficient and internationally comparable assessment of the pre- and postoperative findings of patients with primary non-syndromic craniosynostosis. The additional survey of quality of life provides a valuable contribution to the analysis of affected patients.

## Graphical abstract

**Figure:**
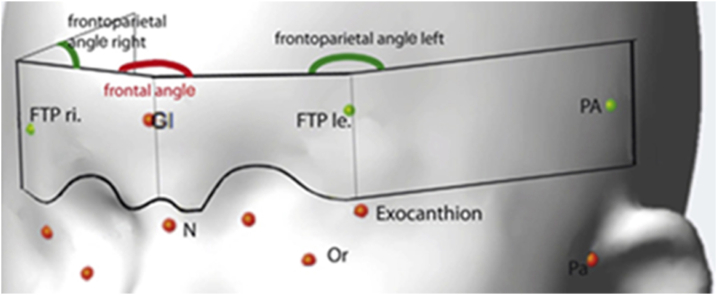

## 1 Introduction

### 1.1 Consequences of craniosynostosis

The development of the child's skull is a complex process. Disorders can lead to premature closure of one or more cranial sutures.^1^ This premature ossification of the cranial sutures, known as craniosynostosis, can lead to skull deformities and a narrowing of the cranial cavity.^1^ The earlier suture ossification occurs, the greater the extent of disruption of physiological growth and development of the neuro- and viscerocranium and the more severe the expected functional impairments.^1^^,^^2^ In addition, the discrepancy between increasing brain volume and restricted intracranial space can lead to increased intracranial pressure with cerebrospinal fluid circulation and cerebral blood flow disorders.^1^, ^2^, ^3^ Clinical consequences can include neurological symptoms (restlessness, frequent crying, sleep disorders, vomiting, optic nerve damage with deterioration in visual acuity and even blindness) and psychomotor developmental disorders.^1^^,^^2^

In the viscerocranial region, growth restriction is manifested by insufficient anterocaudal growth rotation of the maxilla, resulting in midface hypoplasia, hypertelorism, exophthalmos and insufficient expansion of the nasopharynx with chronic respiratory infections and inhibition of food intake with consecutive failure to thrive and developmental disorders or delays.^1^^,^^2^ Increased intracranial and intraocular pressure are an indication for surgical correction.^3^

### 1.2 Treatment of craniosynostosis

Ideally, surgery should be performed before the end of the first year of life.^4^ Severe forms of craniosynostosis, in which several sutures are affected, often even require earliest possible surgical intervention in order to prevent these functional sequelae and to avoid permanent impairment of the external, aesthetic appearance with the stigmatisation that often accompanies it.^1^, ^2^, ^3^, ^4^

In order to be able to initiate early therapy, a detailed and interdisciplinary anamnesis and reliable diagnostics should be carried out (ophthalmologist, neuropaediatrics, ENT, maxillofacial surgery, radiology).^1^^,^^2^^,^^4^ Imaging procedures include sonography of the cranial sutures, X-ray of the skull in 2 planes (p.a. and lateral) and, in complex cases, computer tomography.

It is important to differentiate and distinguish these anomalies from the more common, position-related cranial deformities, as these do not require surgical treatment.^5^

### 1.3 Aim of the present study

Craniosynostoses have already been well studied in terms of aetiology, pathogenesis, symptoms and epidemiology.^1^^,^^2^^,^^6^ However, to our knowledge, scientific studies on the quality of life of such patients are rare. Therefore, the present study aims to present the postoperative results of patients with primary non-syndromic craniosynostosis with regard to symmetry, aesthetics and quality of life. With the aim of further optimizing the care of patients with non-syndromic craniosynostosis, we have developed and describe here a novel, clinically practicable and detailed method of measurement in which pre- and postoperative findings can be assessed simply, objectively and in an internationally comparable manner. It can also be used reliably by inexperienced examiners for diagnostics and therapy planning as well as for growth monitoring. The additional assessment of quality of life provides a valuable contribution to the analysis of the patients concerned.

## 2 Material und method

### 2.1 Ethics

The present study was positively evaluated by the Ethics Committee (Ethics vote: 19–1173_1). Written informed consent from all patients who underwent surgical treatment and their parents was gained.

### 2.2 Patient selection

From 2000 till 2022, 76 patients with primary non-syndromic craniosynostosis were followed up. The patients with craniosynostosis were further subdivided into patients with scapho-, trigono- and anterior plagiocephalus. 47 healthy patients without craniosynostosis formed the control group. All patients were treated by one experienced surgeon at a specialist centre with sophisticated strategies for operational correction. The patients were approximately between 3 and 16 months of age and were excluded if the primary surgery was not performed at our centre.

### 2.3 Study methodology

Standardised photos were taken from all patients preoperative, within a period of 6 weeks after the operation, as well as 6 and 12 months after the operation and then annually until the 5th postoperative year and 10 and 15 years postoperatively. Additional x-ray-imaging was performed for patients with trigono- and anterior plagiocephalus preoperative and within a period from 6 weeks and between 4 and 7 months postoperativly (on average 5 months postoperatively). For detailed information, please refer to Table 1 and to the supplementary information material.

**Table 1:** Pre- and postoperative significant findings (mean value) of patients with different craniosynostoses compared to healthy patients without craniosynostoses (control group).

Craniosynostosis	Scaphocephalus	Trigonocephalus	Anterior Plagiocephalus
**Caliper**			
Cranial length (in cm)	20 (∗)	17	17
Cranial width (in cm)	12 (∗)	15	15
Cranial Index (CI) calculated for all slices	70 (∗)	<90	<90
Diagonal A and Diagonal B (in cm)	≤3.5	≤3.5	6 (∗)
**3-D-Photo**			
frontal angle (in degrees°)	153	130 (∗)	140 (∗)
Fronto-parietal angle (right and left in degrees°)	138	130 (∗)	134 (∗)
Quadrant volume Q1 and Q2 (in ml) estimated over all 11 planes	590	400 (∗)	500 (∗)
Anterior Asymmetry Ratio (ASR)	1	1	0.8 (∗)
**X-ray image** (frontal view)			
MO-MO	12	10 (∗/∗∗)	12
X-ray image (lateral view, p.a.)			
ECA-ECA	20 (∗)	17	17
Nas-Tub	10 (∗)	7	7
EU-EU/MO-MO	8	10 (∗/∗∗)	6
ECA-ECP/ECS-BA	1.1 (∗/∗∗)	1.3	1.3
Inter-MO	14	10 (∗/∗∗)	14
Inter-EU	130 (∗)	120	120
ECA-ECP	170 (∗)	160	160

∗(preoperative significant to control group); ∗∗(directly post-operative significant to control group).

### 2.4 Surveying system

**Fig. 1:**
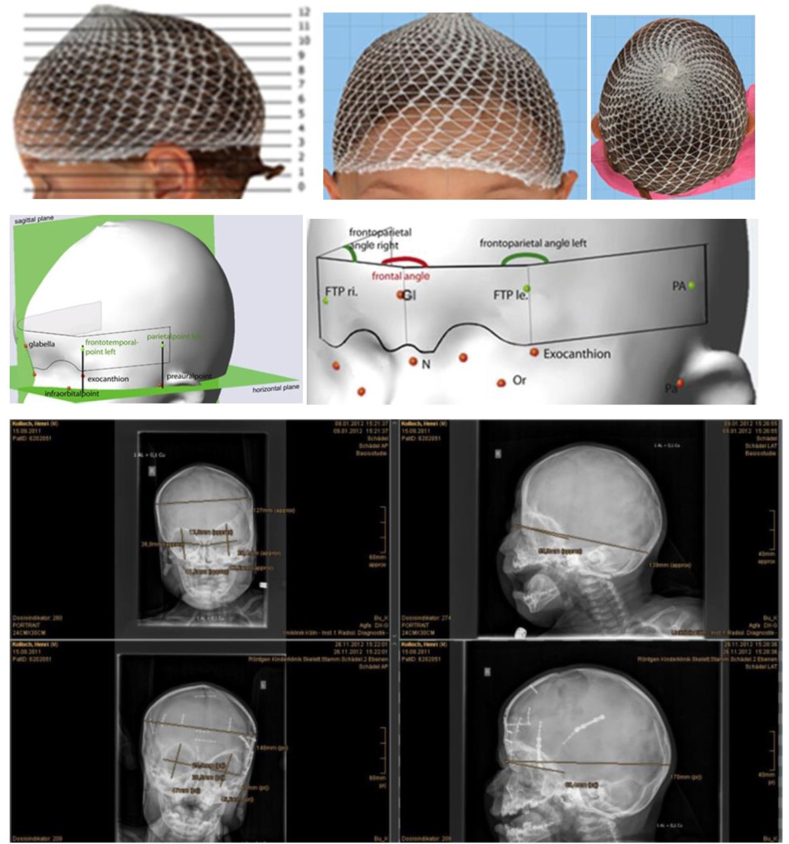
**a-i)** 3-D photometry with soft tissue points and pre- and postoperative X-ray image of a patient with subtle trigonocephalus; a) View of the head with mesh cap for smoothing the scan surface from the lateral left, subdivision of the head into 12 layers; b) View of the head with mesh cap for smoothing the scan surface from the front; c) top view of the head with mesh hood for smoothing the scan surface; d) 3-D photometry with soft tissue points in the view from the lateral left with sagittal and horizontal plane (green), glabella (red), infraorbital point, frontotemporal point (green), exocanthion, parietal point (green) and preaural point marked; e) 3-D photometry with soft tissue points and angles in the view from the frontal left, FTP ri and le: frontotemporal point right and left (green); Gl: glabella (red); N: nasion (red); Or: infraorbital point (red); Pa: preaural point (red); PA: parietal point (green); frontal angle (red); frontoparietal angle right and left (green). The frontal angle was measured between the nasion and the lateral edges of the orbit. The frontoparietal angles were measured on the right and left between the lateral edge of the orbit, the nasion and a fixed parietal point, approx. 1 cm above the base of the ear. f) Preoperative X-ray image in the frontal view (p.a.) with measurement points marked (maximum skull width, interorbital distance, orbital height/width). g) Preoperative X-ray image in the lateral view with marked measuring points (maximum skull length (nasion-inion), length of the anterior cranial fossa (sella-nasion)). h) Post-operative X-ray image in frontal view (p.a.) with osteosynthesis material in place after frontoorbital advancement (FOA) and measurement points marked (maximum skull width, interorbital distance, orbital height/width). i) postoperative X-ray image in the lateral view with osteosynthesis material in place after frontoorbital advancement (FOA) and measurement points marked (maximum skull length (nasion-inion), length of the anterior cranial fossa (sella-nasion)). (For interpretation of the references to colour in this figure legend, the reader is referred to the Web version of this article.)

A novel digital measurement tool was used, that was integrated into a clinically established programme (Cranioform Analytics 3.0, Cranioform AG, Industriestraβe 23, Alpnach, Schweiz®). In addition to manual skull measurement using a caliper, it was possible to set manually defined measuring points on pre- and postoperative x-ray-imaging and 3-D-photographs of the head as part of routine clinical diagnostics. In order to measure each image to scale, an individual distance calibration was first performed. Since the comparison of the measured values takes place within a photo, the calibration remains constant and independent of the recording technique of the photo. Established soft tissue and bone measurement points/landmarks were used and supplemented with various specially defined landmarks as well as detailed symmetry and aesthetic indices.^7^^,^^8^ A total of 64 values were collected per patient and an anatomical reference system/coordinate system was created. Its centre was defined on a connecting line between the points “tragus right” and “tragus left” as well as between the points “lateral canthus right” and “lateral canthus left”. Both centre points were connected to each other (Y-axis). The X-axis was defined by the two centre points and the subnasal point and is perpendicular to the plane between the “right and left tragus”. The Z-axis is also perpendicular to the X- and Y-axes. The skull has now been divided into 12 layers/levels. Layer 0 lies between the points “Tragus right and left” and “subnasal” and is not used for the calculations/analyses. For detailed information, please refer to Fig. 1 a-I and to the supplementary information material.

### 2.5 Quality of life

In addition, a clinical visual assessment of the scars, satisfaction of patients and their parents with the surgical outcome based on the Whitaker classification and quality of life were evaluated using the standardised questionnaire KID-SCREEN-52® with 5 differently graded answer options.^9^, ^10^, ^11^ For children under the age of 8, the questionnaire was completed by the parents or together with the parents and a corresponding parent questionnaire. From the age of 8, the questionnaire was completed independently by the children and parents. The questionnaire includes 52 questions from 10 categories (physical and psychological well-being, emotions, self-perception, autonomy, relationships with parents/home/peers, social support/acceptance, school environment, financial opportunities).

The results of our own Kidscreen-52®-questionnaire were evaluated in comparison to the Kidscreen-52®European Norm-Data. The average health-related quality of life (HRQOL) is given for each of the 10 categories in the form of a sum score on a scale of 1–100. In addition, the standard deviation and the sum score for the 10th, 25th, 50th, 75th and 90th percentiles are given. The higher the value, the higher the HRQOL.^12^, ^13^, ^14^, ^15^, ^16^

### 2.6 Statistical analysis

The resulting pre-and postoperative values were compared with values of a healthy control group without craniosynostosis of the same age and standard values from the literature.^7^, ^8^, ^17^, ^18^ In order to exclude the pre- and postoperative scans and the associated temporal or physiological changes in growth, the individual age of each patient was taken into account using a statistical analysis of covariance. Postoperative changes in distance were measured in cm and volume were calculated in ml. Statistical analysis was performed using SigmaPlot 13 graphics and statistical software (Systat Software GmbH, Erkrath, Germany). Regression analyses and two-tailed Student's t-tests were performed. A p-value <0.05 is considered statistically significant. A p-value >0.1 as not significant. Additionally, p-values of <0.1 are considered mildly significant and p-values of <0.01 were considered highly significant.

## 3 Results

### 3.1 Patient category

Of the total of 76 patients, 35 patients had scaphocephalus, 34 patients had trigonocephalus and 8 patients had anterior plagiocephalus.

Of the patients diagnosed with scaphocephalus, 29 patients were male and 6 patients were female. Among the patients diagnosed with trigonocephalus, 24 patients were male and 10 patients were female. Of the patients diagnosed with anterior plagiocephalus, the left coronal suture was affected in 4 patients, 2 of whom were male and 2 female. In 3 patients, the right coronal suture was affected; 1 patient was male and 2 patients were female.

Of the 47 healthy patients in the control group without craniosynostosis, 34 patients were male and 13 patients were female.

All patients had an age between 3 months and 18 years.

### 3.2 Preoperative findings

Preoperatively, all measurement methods (caliper, X-ray and 3D photo) showed a statistically significant higher value (p < 0.05) for determining the cranial length and a statistically significant lower value for determing the cranial width in patients with a diagnosis of non-syndromic scaphocephalus and a statistically significant lower value for determining the anterior cranial fossa in all patients with a diagnosis of non-syndromic trigonocephalus and anterior plagiocephalus (diagonal A and diagonal B, anterior left and right quadrant volumes Q1 and Q2, anterior asymmetry ratio (ASR), frontal angle, right and left fronto-parietal angle)).

The measured values of the orbital region to characterize the hypotelorism with temporal retraction and parietal protrusion typically occurring in trigonocephalus were also statistically significant lower preoperatively (p < 0.05) than the measured values of the healthy control group without trigonocephalus.

All other measured values corresponded to the standard values/normal values from the literature.

None of the patients showed clinical or radiological evidence of increased intracranial pressure or neurological abnormalities or signs of developmental disorders.

### 3.3 Postoperative findings

#### 3.3.1 Esthetic appearance, surgical procedure

No major postoperative complications occurred. All patients were categorized as Whitaker I postoperatively, so that no further treatment was necessary or desired.

For patients with scaphocephalus, we performed the procedure of total vertex craniectomy (approximately 9 cm wide) including the sagittal suture as well as the proximal coronal and lambdoid sutural complex. Mean age at the time of surgery was 5.1 months (ranging from 3 to 12 months). The mean operation time for total vertex craniectomy was 95 min (range from 49 to 281 min).

Surgical reconstruction of trigonocephalus and plagiocephalus was performed using the standardised frontoorbital advancement (FOA). The mean age at the time of the FOA was 9.6 months (range from 6 to 16 months). The mean operation time for patients with trigonocephalus was 148 min (range from 53 to 241 min) and for patients with plagiocephalus 153 min (range from 95 to 273 min).

The average hospital stay after operation was 6 days for patients with scapho- and trigonocephalus and 3 days for patients with plagiocephalus.

##### 3.3.1.1 Cranial width and anterior cranial fossa

The first postoperative measurements showed a statistically significant increase (p < 0.05) in the values for determining the cranial width in patients with a diagnosis of non-syndromic scaphocephalus and for determining the anterior cranial fossa in all patients with a diagnosis of non-syndromic trigonocephalus and anterior plagiocephalus (diagonal A and diagonal B, anterior left and right quadrant volumes Q1 and Q2, anterior asymmetry ratio (ASR), frontal angle, right and left fronto-parietal angle). During the following course of growth these and all measurements in this area remained close to, but consistently lower than the (standard) values reported in the literature. for healthy patients without craniosynostosis.

The average intraoperative displacement distance for the frontal orbital advancement (FOA) (measured with the calliper) corresponded to the average pre- and postoperative measurement results (calliper, X-ray images or 3D photos).

##### 3.3.1.2 Orbital region

The measured values of the orbital region, to characterize the hypotelorism with temporal retraction and parietal protrusion typically occurring in trigonocephalus, initially remained the same on average immediately postoperatively and thus statistically significant lower (p < 0.05) than the measured values of the healthy control group without trigonocephalus. In the course of growth up to the average age of 13 years, these measured values approached the average values of the healthy control group without trigonocephalus or the measured values reported in the literature/the norm, but always remained lower. In addition, as in the literature, the measured values remained more or less constant with increasing age.

Overall, all measurements based on the 3D photographs showed less variability and smaller standard deviations (even from the average values of healthy patients without craniosynostosis or the normal values) than the measurements based on the X-ray images and with the callipers. Compared to the calliper and radiograph-based measurements, the 3D photography-based values were consistently slightly higher. The measurements based on X-ray images and callipers showed similar average values with a similar variability or standard deviation.

##### 3.3.1.3 Quality of life

The evaluation of the Kidscreen-52-questionnaires® showed that the quality of life of patients who had undergone surgery tended to be higher than that of healthy patients in the control group without craniosynostosis and the European Normdata Kidscreen-52 Sumscore. Only in the Social Support & Peers category was the average sum score lower than that of the healthy control group and the European Normdata Kidscreen-52 Sumscore ® (for detailed information, please refer to Table 1 and to the supplementary information material).

## 4 Discussion

### 4.1 Strengths

Overall, the new 3D measurement technique with the specified indices based on photographs is better suited for an objective result, quality and long-term control than X-ray control, as it is more detailed and, due to the lower scatter and standard deviation of the measured values, also more reliable and reproducible than the evaluation of X-ray images. An additional advantage in line with statements in the literature is the absence of radiation as well as its simple and therefore effective clinical applicability, especially for the inexperienced examiner.^19^^,^^20^ Thanks to automatic interfaces to other clinically established programmes, such as X-ray, the results can be used in an interdisciplinary manner for continuous growth monitoring.

As noted above, the calculation of the skull length and width and the cranial index (CI) is particularly suitable for visualizing and analysing the postoperative changes in patients with primary, non-syndromic scaphocephalus.^20^

The values Q1 and Q2 are especially suitable for illustrating the anterior volume gain resulting postoperatively in patients with a primary, non-syndromic trigonocephalus.^20^ The calculation of the anterior and posterior symmetry ratio is really relevant for the visualization of postoperative changes in patients with primary, non-syndromic anterior and posterior plagiocephalus.^20^ Anterior symmetry ratio (ASR) illustrated the ratio of the higher anterior volume to the lower anterior volume and showed a gain in symmetry for anterior non-syndromic plagiocephaly, independent of the affected side.^20^ Posterior Asymmetry Ratio (PSR) illustrated the higher posterior volume to the lower posterior volume and is applicable for lambdoid synostosis.^20^ The measured values for both come close to the perfect value of 1^20^. Surgical correction of patients diagnosed with trigonocephaly is a major challenge due to the complex development of the skull. The overall very good measurement results with approximation to the standard/normal values from the literature as well as the very good evaluations using the Whitaker score and Kidscreen 52 questionnaire prove that the surgical procedure using FOA normalizes the intracranial volume on the one hand and, on the other, restores a symmetrical and aesthetically pleasing appearance by remodelling the frontoorbital bone.

As the Whitaker score reflects a subjective assessment, we consider the use of objective and reliably reproducible measured values and indices as well as a questionnaire specially tailored to the children and parents concerned to be particularly important and meaningful.

### 4.2 Limitations

The study was conducted on a relatively small sample, which potentially limits the generalisability of the results and ability to extrapolate meaningful conclusions across different populations or healthcare settings. In addition, the fact that only one surgeon performed the operations could also lead to distortions in the results, and may lead to potential bias. Although the consistency of the novel measurement system is assessed over a long-term follow-up period of up to 15 years, more modern methods and other more advanced digital or AI-based techniques would detect more subtle changes over even longer periods of time. It should be noted that there are also technical limitations associated with the use of the measurement tool. It requires a potential training and learning curve as well as specific hardware and software for implementation. In addition, inexperienced users may face challenges during implementation. Therefore further multicenter validation studies with a larger sample size and other surgical teams with different levels of expertise should be conducted to confirm the present results. Furthermore the results should also be compared with other advanced digital or AI-based techniques and modern alternatives in the future.

Finally it should be noted, the use of the Kidscreen 52 questionnaire as a subjective assessment is a strength, but cultural or demographic factors can also influence these results.

## 5 Conclusions

3D photogrammetry with the novel measurement technique and the specified indices has great potential in the future, especially in conjunction with the possible use of artificial intelligence. The fact that it is radiation-free and easy to use clinically, even for inexperienced examiners, offers considerable advantages for tracking the clinical course of patients with diagnosed and surgically corrected craniosynostosis in an objective, detailed, reliable and interdisciplinary manner.

## Patient's/Guardian's consent

Written informed consent from all patients who underwent surgical treatment and their parents was gained.

## Ethical clearance

The present study was positively evaluated by the Ethics Committee of the Medical Faculty of the University of Cologne. Ethics vote: 19–1173_1.

Written informed consent from all patients who underwent surgical treatment and their parents was gained.

## Sources of funding

Funding information: This research did not receive any specific grant from funding agencies in the public, commercial, or not-for-profit sectors.

## Declaration of competing interest

The authors declare that they have no known competing financial interests or personal relationships that could have appeared to influence the work reported in this paper.
