# Supplementary material for: Novel digital measurement system for predicting surgical outcomes in patients with primary non-syndromic craniosynostosis
Source: J Oral Biol Craniofac Res. 2025 Feb 21;15(2):406–11. doi: 10.1016/j.jobcr.2025.01.025 (PMC11904546; doi:10.1016/j.jobcr.2025.01.025)
Supplement: Multimedia component 1 [file mmc1.docx]

**Supplemental Tables**

**Supplemental Table 1:**

Pre- and postoperative findings of patients with different craniosynostoses compared to healthy patients without craniosynostoses (control group)

| **Diagnosis** | **Craniosynostosis** | **Scaphocephalus** | **Trigonocephalus** | **anterior plagio-**  **cephalus (left coronal suture affected)** | **anterior plagio-**  **cephalus (right coronal suture affected)** | **Healthy patients without craniostenosis (control group)** |
| --- | --- | --- | --- | --- | --- | --- |
| total | 76 | 35 | 34 | 4 | 3 | 47 |
| male | 56 | 29 | 24 | 2 | 1 | 34 |
| female | 20 | 6 | 10 | 2 | 2 | 13 |
| OP technique |  | Total vertex  craniotomie | Standardised fronoorbital advancement (FOA) | Standardised fronoorbital advancement (FOA) | Standardised fronoorbital advancement (FOA) |  |
| age range of the patients (minimum-maximum) | 3 months – 18 years | 3 months – 18 years | 3 months – 18 years | 3 months – 18 years | 3 months – 18 years | 3 months – 18 years |
| Age at the time of surgery (minimum-maximum) | 3 – 16 months | 3 – 12 months  (mean age 5.1 months) | 6-16 months  (mean age 9.6 months  with a standard deviation of 1.9 months) | 6-16 months  (mean age 9.6 months  with a standard deviation of 1.9 months) | 6-16 months  (mean age 9.6 months  with a standard deviation of 1.9 months) |  |
| Patients with a family history of predisposition | 4 | - | 4 | **-** | **-** |  |
| Average preoperative weight (minimum-maximum) | 8.55kg  (8.2kg – 8.9kg) | 7.5kg  (5.4-10kg) | 8.8kg  (6.7 – 10.6kg) | 8.55kg  (8.2kg – 8.9kg) | 8.55kg  (8.2kg – 8.9kg) | 8.55kg  (8.2kg – 8.9kg) |
| Average size preoperative (minimum-maximum) | 71cm (70-72cm) | 71cm (70-72cm) | 73.1cm (62-84cm) | 71cm (70-72cm) | 71cm (70-72cm) | 71cm (70-72cm) |
| Preoperative clinical symptoms (neurological disorders, congestive papilla, optic  atrophy/ visual deterioration/ atrophy hearing disorders) | no | no | no | no | no | **-** |

| **Diagnosis** | **Craniosynostosis** | **Scaphocephalus** | **Trigonocephalus** | **anterior plagio-**  **cephalus (left coronal suture affected)** | **anterior plagio-**  **cephalus (right coronal suture affected)** | **Healthy patients without craniostenosis (control group)** |
| --- | --- | --- | --- | --- | --- | --- |
| Radiological symptoms pre-operative (cloud skull) | no | no | no | no | no |  |
| Preoperative hemoglobin (minimum-maximum) | 11,75 g/dl  (9,5-14 g/dl) | 11.5g/dl  (9-13.5 g/dl) | 11,75 g/dl  (9,5-14 g/dl) | 11,75 g/dl  (9,5-14 g/dl) | 11,75 g/dl  (9,5-14 g/dl) | 11,75 g/dl  (9,5-14 g/dl) |
| Postoperative hemoglobin (minimum-maximum) | 9.7g/dl (7.4-13.5 g/dl) | 9.7g/dl (7.4-13.5g/dl) | 9.7g/dl (7.4-13.5g/dl) | 9.7g/dl (7.4-13.5g/dl) | 9.7g/dl (7.4-13.5g/dl) | **-** |
| Mean duration of the surgical procedure  (minimum-maximum) |  | 95 min (49–281 min) | 148 minutes  (53-241 minutes) | 153 minutes  (95-273 minutes) and the median duration 143 minutes with a standard deviation of 44 minutes | 153 minutes  (95-273 minutes) and the median duration 143 minutes with a standard deviation of 44 minutes | - |
| Average displacement distance in the area of the temporal fossa  (minimum-maximum) |  | - | 8.7mm (7-16mm) | **-** | **-** | **-** |
| average blood loss (minimum-maximum) |  | 430ml (200 – 1000ml) | 430ml (200 – 1000ml) | 430ml (200 – 1000ml) | 430ml (200 – 1000ml) | **-** |
| mean amount of erythrocyte concentrates (EC)  (minimum-maximum) | **-** | 265 ml (50–525 ml) | 265 ml (50–525 ml) | 265 ml (50–525 ml) | 265 ml (50–525 ml) | **-** |
| mean amount of fresh frozen plasma (FFP)  (minimum-maximum) | **-** | 240 ml (42.50–500 ml) | 240 ml (42.50–500 ml) | 240 ml (42.50–500 ml) | 240 ml (42.50–500 ml) | **-** |
| Patients necessitating further transfusion of blood com-ponents |  | 9 | 8 | 1 | 1 | **-** |
| Intensive care unit stay  (days postop.) | 1 | 1 | 1 | 1 | 1 | **-** |
| Postoperative complication/ reoperation | no | no | no | no | no | **-** |

| **Diagnosis** | **Craniosynostosis** | **Scaphocephalus** | **Trigonocephalus** | **anterior plagio-**  **cephalus (left coronal suture affected)** | **anterior plagio-**  **cephalus (right coronal suture affected)** | **Healthy patients without craniostenosis (control group)** |
| --- | --- | --- | --- | --- | --- | --- |
| Average stay on normal ward (days postop.)  (minimum-maximum) |  | 6.3 days (4 – 8 days) | 6.3 days (4 – 8 days) | 3 | 3 | **-** |
| Whitaker- classification (postop.) | I | I | I | I | I | **-** |
| Average time until the X-ray check (postop.)  (minimum-maximum) | within 6 weeks and  4-7 months | **-** | within 6 weeks and  4-7 months  (4.9 months) | within 6 weeks and  4-7 months  (4.9 months) | within 6 weeks and  4-7 months  (4.9 months) | **-** |
| Age at follow-up examination (minimum-maximum) | 3 months – 18 years | 3 months – 18 years | 6 months – 18 years | 6 months – 18 years | 6 months – 18 years | 3 months – 18 years |
| Time of 3-D photometry | - pre-operative - within 6 weeks postop. - after 6 and 12 months postop. - then annually up to the age of 5 years - with 10 and 15 years postop. | - pre-operative - within 6 weeks postop. - after 6 and 12 months postop. - then annually up to the age of 5 years - with 10 and 15 years postop. | - pre-operative - within 6 weeks postop. - after 6 and 12 months postop. - then annually up to the age of 5 years - with 10 and 15 years postop. | - pre-operative - within 6 weeks postop. - after 6 and 12 months postop. - then annually up to the age of 5 years - with 10 and 15 years postop. | - pre-operative - within 6 weeks postop. - after 6 and 12 months postop. - then annually up to the age of 5 years - with 10 and 15 years postop. | - with 5 months - with 16 months - with 24 months - annually up to the age of 5 years - with 10 and 15 years |
| Postoperative develop-mental disorders / neurological symptoms | no | no | no | no | no | **-** |

**Supplemental Table 2:**

Example for each measurement of the 3D photos and the X-ray images (frontal and lateral view) pre- and postoperatively with collection of various skull measurements between the defined anthropometric points/landmarks (distances (in cm), volume (in ml), circumference, angle (in degrees°) with the respective minimum and maximum value, the mean value, the standard deviation, the significance and the comparison with the data from the literature

3-D-Photo

| **point/Value** | **Discription** | **Mini-mum** | **Maxi-mum** | **Mean** | | **standard deviation (SD)** | **Mean value from the literature/ standard value** | | **Significance (*)** |
| --- | --- | --- | --- | --- | --- | --- | --- | --- | --- |
| Gl | Glabella |  |  |  | |  |  | |  |
| N | Nasion |  |  |  | |  |  | |  |
| FTP | frontotemporal point |  |  |  | |  |  | |  |
| Exocanthion | soft tissue point located at the outer commissure of each eye fissure |  |  |  | |  |  | |  |
| Or | Infraorbital point |  |  |  | |  |  | |  |
| PA | Parietal point, approx. 1 cm above the base of the ear |  |  |  | |  |  | |  |
| Pa | Preaural point |  |  |  | |  |  | |  |
|  |  |  |  |  | |  |  | |  |
| Cranial length (in cm) | For the middle plane (level 5), the particular length through the midpoint was determined | 16.5 | 20 | 18.2 | | 0.97 | 18.4 | | *(preoperative for scaphocephalus) |
| Cranial width (in cm) | length of the x axis through the midpoint at level 5 | 13.6 | 16.4 | 14.8 | | 0.73 | 17.2 | | *(preoperative for scaphocephalus) |
| Diagonal A and Diagonal B | The longest diagonal of the right side of the head versus the analogous diagonal on the left side. The appropriate measuring track was automatically explored in 5 steps from 15 to 80 from the x axis to find the longest diagonal | 5.8  3.1 | 12.4  8.66 | 9.10 (preop.)  5.88 (postop.) | | 3.29  2.78 | < 3,5 | | *(preoperative for anterior plagiocephalus) |
| Quadrant volume Q1 (in ml) estimated over all 11 planes | volume of the anterior left cranial quadrant | 410 | 832.6 | 584 | | 101.51 | - | | *(preoperative for trigono-and anterior plagiocephalus) |
| Quadrant volume Q2 (in ml)  estimated over all 11 planes | volume of the right anterior quadrant | 467.8 | 821 | 605.8 | | 97.23 | - | | *(preoperative for trigono-and anterior plagiocephalus) |
| Quadrant volume Q3 (in ml) estimated over all 11 planes | volume of the posterior right quadrant | 406.4 | 699.6 | 529.6 | | 62.81 | - | |  |
| Quadrant volume Q4 (in ml) estimated over all 11 planes | volume of the posterior left quadrant | 422.9 | 652.5 | 523.9 | | 58.3 | - | |  |
| Anterior Asymmetry Ratio (ASR) | ratio of the higher anterior volume to the lower anterior volume ASR1⁄4 Qmax/Qmin | 0.8 | 1 | 0.932 | | 0.06 | 1 | | *(preoperative for anterior plagiocephalus) |
| Posterior Asymmetry Ratio (PSR) | ratio of the higher posterior volume to the lower posterior volume PSR1⁄4 Qmax/Qmin | 0.8 | 1 | | 0.927 | 0.05 | 1 |  | |
| Cranial Index (CI) calculated for all slices | ratio of the maximum head width versus the maximum head length (Cranial width/cranial length x 100) | 72 | 88.7 | | 81.4 | 3.94 | <90 | *(preoperative for scaphocephalus) | |
| frontal angle in  degrees° | Angle between the nasion and the lateral edges of the orbit | 129.6 | 153.6 | | 143.1  Median = 142.3 | 5.5 | 153.3 | *(preoperative for trigono-and anterior plagiocephalus) | |
| Standard values from the literature |  | 135 | 153 | | 144.7  Median = 146 | 5.5 | 153.3 |  | |
| Fronto-parietal angle  (right) in degrees° | right angle  between the lateral edge of the orbit, the nasion and a fixed parietal point, approx. 1 cm above the base of the ear | 126.3 | 137.2 | | 134.3  Median = 134.7 | 2.7 | 137.1 | *(preoperative for trigono-and anterior plagiocephalus) | |
| Standard values from the literature |  | 130 | 144 | | 137.1  Median = 137 | 4.6 | 137.1 |  | |
| Fronto-parietal angle  (left) in degrees° | left angle  between the lateral edge of the orbit, the nasion and a fixed parietal point, approx. 1 cm above the base of the ear | 128.2 | 141.2 | | 134.4  Median = 133.9 | 3.5 | 139 | *(preoperative for trigono-and anterior plagiocephalus) | |
| Standard values from the literature |  | 133 | 145 | | 139  Median = 139 | 4.5 | 139 |  | |

X-ray image (frontal view)

| **point/Value** | **Discription** | **Mini-mum** | **Maxi-mum** | **mean** | **standard deviation (SD)** | **Mean value from the literature / standard value** | **Significance (*)** |
| --- | --- | --- | --- | --- | --- | --- | --- |
| MO | innermost point of the medial orbit |  |  |  |  |  |  |
| LO | outermost point of the lateral orbit |  |  |  |  |  |  |
| EU | Euryon = most lateral point of the neurocranium |  |  |  |  |  |  |
| LOSP | Intersection of the lateral orbit with the sphenoid wing |  |  |  |  |  |  |
| MO-MO | Interorbital distance; determination of the minimum distance of the med. orbital boundary |  |  | 12.2 | 2 |  | *(pre- and directly post-operative for trigono- cephalus) |
| LO-LO | Distance between the most lateral points of the orbitae;  determination of the maximum distance of the lat. orbital boundary |  |  | 20,0 | 2 |  |  |
| EU-EU | Maximum width of the neurocranium; Max. distance skull  (here: line through upper orbit) | 13.7 | 13.9 | 13.8 | 0.8 |  |  |
| Losp-Losp | Distance between the intersection points of the lateral orbit and the sphenoid wing;  Determination of the distance between overlaps pterion / lat. orbital wall |  |  |  |  |  |  |

**X-ray image (lateral view, p.a.)**

| **point/Value** | **Discription** | **Mini-mum** | **Maxi-mum** | **mean** | **standard deviation (SD)** | **Mean value from the literature / standard value** | **Significance (*)** |
| --- | --- | --- | --- | --- | --- | --- | --- |
| ECA | Anterior point of the neurocranium |  |  |  |  |  |  |
| ECP | Most distal point of the neurocranium |  |  |  |  |  |  |
| ECS | Highest point of the neurocranium |  |  |  |  |  |  |
| BA | Basion = most posterior and deepest point of the anterior edge of the foramen magnum |  |  |  |  |  |  |
| Nas | Nasion = foremost point of the sutura fronto-nasalis |  |  |  |  |  |  |
| Tub | Tuberculum sellae = highest point of the Sella turcica |  |  |  |  |  |  |
| ECA-ECA | Length of the neurocranium | 17 | 17.3 | 17.2 | 1.15 |  | * (preoperative for scaphocephalus) |
| ECS-BA | Distance between basion and neurocran.sup. =  Height of the neurocranium | 13 | 13.2 | 13.1 | 0.9 |  |  |
| Nas-Tub | Length of the anterior cranial fossa |  |  |  |  | 7.2 | * (preoperative for scaphocephalus) |
| EU-EU/LO-LO (V) | Preoperative growth ration = relation between the width of the neurocranium and the distance between the lateral points of the orbit |  |  | 1.64 |  | - |  |
| EU-EU/LO-LO (P) | postoperative growth ration = relation between the width of the neurocranium and the distance between the lateral points of the orbit |  |  | 1.6 |  | 1.67 |  |
| LO-LO/MO-MO (V) | preoperative growth ratio = distance relationship between the most lateral points of the orbitae and the interorbital distance |  |  | 5.93 |  | - |  |
| LO-LO/MO-MO (P) | postoperative growth ratio = distance relationship between the most lateral points of the orbitae and the interorbital distance |  |  | 6.27 |  | 4.1 |  |
| EU-EU/MO-MO (V) | preoperative growth ration = relation between the width of the neurocranium and the interorbital distance; measure of hypotelorism |  |  | 9.74 |  | - | *(for trigonocephalus) |
| EU-EU/MO-MO (P) | postoperative growth ration = relation between the width of the neurocranium and the interorbital distance; measure of hypotelorism |  |  | 10.10 |  | 6.9 | *(directly post-operative for trigonocephalus) |
| ECA-ECP/ECS-BA (V) | preoperative growth ratio = relation between length and height of the neurocranium; corresponds to cranial index (CI) |  |  | 1.22 |  | - | *(for scapho-cephalus) |
| ECA-ECP/ECS-BA (P) | postoperative growth ratio = relation between length and height of the neurocranium; corresponds to cranial index (CI) |  |  | 1.22 |  | 1.3 | *(for scapho-cephalus) |
| CI (V) | Cranial index (preoperative) = ratio of max. width to max. length of the neurocranium |  |  | 71 |  | <85 | *(for scapho-cephalus) |
| CI (P) | Cranial index (postoperative) = ratio of max. width to max. length of the neurocranium |  |  | 81 |  | <85 |  |

Pre- and postoperative relations

| **point/Value** | **Discription** | **Mini-mum** | **Maxi-mum** | **mean** | **standard deviation (SD)** | **Mean value from the literature / standard value** | **Significance (*)** |
| --- | --- | --- | --- | --- | --- | --- | --- |
| Inter-MO (V) | Preoperative ratio inter MO | 10.7 | 19.7 | 13.56 | 2.37 | 18.6 | *(for trigonocephalus) |
| Inter-MO (P) | Postoperative relationship between MO | 11.3 | 22.4 | 14.85 | 3.07 | 18.2 | *( directly post-operative for trigonocephalus) |
| Inter-LO (V) |  | 74.7 | 90.1 | 80.47 | 4.55 | 72.9 |  |
| Inter-LO (P) |  | 79.9 | 100.0 | 86.91 | 6.3 | 74.7 |  |
| Inter-EU V | preoperative distance between the maximum width of the neuro-cranium; Max. distance skull (here: line through upper orbit) | 114.0 | 147.0 | 132.14 | 9.62 | 116.8 | *(preoperative for scaphocephalus) |
| Inter-EU P | Postoperative distance between the maximum width of the neuro-cranium; Max. distance skull (here: line through upper orbit) | 121.0 | 159.0 | 139.93 | 9.77 | 124.8 |  |
| Inter-Losp V | preoperative distance between the intersection points of the lateral orbit and the sphenoid wing | 67.6 | 88.3 | 76.36 | 5.73 | 96.9 |  |
| Inter-Losp P | postoperative distance between the intersection points of the lateral orbit and the sphenoid wing | 73.4 | 92.2 | 81.77 | 6.37 | 101.7 |  |
| Nas-TUB V |  | 48.1 | 57.8 | 52.17 | 2.91 | - |  |
| Nas-TUB P |  | 47.7 | 63.7 | 56.26 | 4.49 | - |  |
| ECA-ECP V | preoperative distance between the furthest anterior and posterior point | 146.0 | 188.0 | 165.36 | 13.64 | 152.2 | *(for scaphoocephalus) |
| ECA-ECP P | postoperative distance between the furthest anterior and posterior point | 160.0 | 196.0 | 172.79 | 10.41 | 163.3 |  |
| ECS-BA V | Preoperative distance between the highest point and the Basion | 120.0 | 146.0 | 135.07 | 8.81 | 114 |  |
| ECS-BA P | Postoperative distance between the highest point and the Basion | 132.0 | 152.0 | 142.21 | 6.22 | - |  |
| Inter-Losp-Losp/ Inter-EU | Ratio between the distance between the intersection points of the lateral orbit and the sphenoid wing and the distance between the maximum width of the neurocranium; Max. distance skull (here: line through upper orbit) measure of temporal retraction in parietal protrusion |  |  |  |  |  | *(pre- and directly post-operative for trigonocephalus) |
| sphen-nas/ nas-lambda | measure of shortening of the anterior cranial fossa |  |  |  |  |  |  |
| Inter Losp and Nasion-Sphen | analogy to the Oi angle (Nasio-Sphen. / Inter Losp) to be expected in trigono smaller |  |  |  |  |  |  |

**Supplemental Table 3:**

Results of our own Kidscreen-52®-questionnaire evaluation in comparison with the Kidscreen-52®European Norm-Data (average 15,726 respondents). The average health-related quality of life (HRQOL) is given for each of the 10 categories in the form of a sum score on a scale of 1-100. In addition, the standard deviation and the sum score for the 10th, 25th, 50th, 75th and 90th percentiles are given. The higher the value, the higher the HRQOL.

|  | own sur-vey |  |  |  |  |  |  |  |  | European  Normdata |  |  |  |  |
| --- | --- | --- | --- | --- | --- | --- | --- | --- | --- | --- | --- | --- | --- | --- |
|  |  |  | Percen-tiles |  |  |  |  |  |  | Percen-  tiles |  |  |  |  |
| **Category** | mean | Standard  deviation (SD) | 10 | 25 | 50 | 75 | 90 | mean | Standard-  Deviation (SD) | 10 | 25 | 50 | 75 | 90 |
| **Physical well-being** | 82.77 | 21.75 | 44.5 | 72.91 | 95 | 95 | 100 | 72.08 | 17.75 | 50 | 60 | 75 | 85 | 95 |
| **Psychological well-being** | 86.55 | 9.44 | 75 | 81.24 | 87.5 | 92.71 | 100 | 74.94 | 15.4 | 54.17 | 66.67 | 75 | 87.5 | 91.67 |
| **Moods & emotions** | 88.48 | 9.34 | 77.14 | 82.14 | 91.07 | 93.75 | 96.43 | 81.38 | 13.39 | 64.29 | 75 | 82.14 | 92.86 | 96.43 |
| **Selfperception** | 90.23 | 11.03 | 66.67 | 87.5 | 95 | 96.25 | 100 | 76.72 | 16.75 | 55 | 65 | 80 | 90 | 95 |
| **Autonomy** | 75.61 | 9.79 | 66.67 | 69.16 | 72.5 | 80 | 93.5 | 75.32 | 18.01 | 50 | 65 | 75 | 90 | 100 |
| **Parent relation & home life** | 84.72 | 16.68 | 76.25 | 79.17 | 87.5 | 95.83 | 98.75 | 77.7 | 15.6 | 58.33 | 66.67 | 79.17 | 91.67 | 95.83 |
| **Social support&peers** | 64.37 | 31.41 | 15.4 | 20.5 | 75 | 85.41 | 99.17 | 67.93 | 18.09 | 45.83 | 58.33 | 70.87 | 79.17 | 91.67 |
| **School environment** | 83.52 | 18.83 | 62 | 75 | 87.5 | 95.83 | 100 | 69.43 | 17.87 | 45.83 | 58.33 | 70.83 | 83.33 | 91.67 |
| **Bullying** | 91.67 | 18.54 | 75 | 91.67 | 100 | 100 | 100 | 88.43 | 15.15 | 66.67 | 83.33 | 91.67 | 100 | 100 |
| **Financial resources** | 85.99 | 19.98 | 57.7 | 81.24 | 91.67 | 100 | 100 | 66.71 | 25.57 | 25 | 50 | 75 | 91.67 | 100 |
